# Evaluation of a program to strengthen general practice care for patients with chronic disease in Germany

**DOI:** 10.1186/s12913-017-2000-2

**Published:** 2017-01-21

**Authors:** Michel Wensing, Joachim Szecsenyi, Christian Stock, Petra Kaufmann Kolle, Gunter Laux

**Affiliations:** 1 Heidelberg University Hospital, Department General Practice and Health Services Research, Marsilius-Arkaden, Turm West, INF 130.3, 69120 Heidelberg, Germany; 2 AQUA Institute for Applied Quality Improvement and Research in Health Care, Göttingen, Germany; 3 Institute of Medical Biometry and Informatics, University of Heidelberg, Heidelberg, Germany

**Keywords:** Chronic disease, Practice management, Primary care, Evaluation research, Health services research

## Abstract

**Background:**

A program to strengthen general practice care for patients with chronic disease was offered in Germany. Enrollment was a free individual choice for both patients and physicians. This study aimed to examine the long-term impact of this program.

**Methods:**

Two comparative evaluations were done, at 4 and 5 years (T1 and T2) after start of the program. In each year, patients in the program were compared with patients in usual care. Measures were based on routinely collected data and concerned 11 aspects of primary care and hospital care. Study groups were compared, using regression analysis adjusted for confounders and clustering.

**Results:**

Data on 1.187.597 and 1.591.017 eligible patients were available for the analysis for T1 and T2, respectively. Compared to usual care, the program was associated with more visits to the GP per patient (adjusted difference at T2: +1.98), more drugs prescribed per patient (+0.071), lower percentage of drugs that should be avoided (−0.699), and lower yearly medication costs per patient (−85.39 euro). The number of referrals to ambulatory specialists, either with or without referral from GP, was reduced at T2. In hospital care, the program was associated with fewer hospital admissions per patient per year (−0.017) and fewer avoidable hospital admissions of all admissions (−1.165%). Total hospital costs were slightly higher in T1, but lower in T2. Days in hospital and number of readmissions were lower at T2 only.

**Conclusion:**

The program has increased the role of general practice in healthcare for patients who chose to be included in the program of intensified general practice care.

## Background

A wide range of programs and policies have been applied to improve healthcare for patients with chronic diseases, often in research or demonstration projects. In Baden-Wuerttemberg, a German federal state with about 10.7 million inhabitants, a program was introduced to strengthen general practice care in the year 2008. This program, which is still running, is targeted at enhancing the role of general practice in healthcare for patients with chronic diseases [1]. It emphasized pro-active, organized and evidence-based management of patient populations. Table 1 presents the program in relation to a framework for high-quality primary care [2].

**Table 1:** Description of the program to enhance general practice care on a framework for high-performing primary care [2]

Components of high-performing primary care	General description	Specific details
1 Engaged leadership	GPCC is arranged in special contracts, which have been developed by organisations of GPs in collaboration with health insurers.	AOK, the largest health insurer in the region, initiated the program together with the regional association of GPs, and supported it over many years.
2 Data-driven improvement	The physician participates in quality circles: small groups of physicians who receive feedback on their prescribing, evidence based information and plan improvements. The practice has a data-orientated quality system and decision support for prescribing medication.	The AQUA-institute, Goettingen, is responsible for the data-based feedback reports for physicians. GPs take part in 4 quality circle meetings per year in which benchmark reports with own prescribing data is discussed under supervision of a trained peer moderator.
3 Empanelment	The physician participates in disease management programs (DMP) which concern panels of patients with diabetes, asthma/COPD, and coronary heart disease.	Partipation by patients is voluntary and based on written informed consent. After consent, patients are added to the panel of patients in GP-centred care.
4 Team-based care	Disease management programs imply enhanced participation of practice assistants in clinical work.	Practice assistants are encouraged to take part in an additional training program (VERAH) for better management of patients with chronic diseases. Practices who have their assistants qualified are entitled to receive a financial bonus.
5 Patient-team partnership	Self-management support is an important component of disease management programs.	Patients who participate in a DMP are offered a validated educational program. This comprises of informative group meetings.
6 Population management	The decisions of the physician on pharmaceutical treatment follow prevailing recommendations, for instance regarding the prescription of discount drugs and such with therapeutic benefit (using a software tool).	Feedback and benchmarking on prescribing is supported by short written evidence reports. Recommendations are strictly evidence-based and not influenced by industry. Prompts in the software of a practice support use of generic and discounted drugs where eligible.
7 Continuity of care	Referrals to medical specialists are preceded by relevant diagnostic procedures and treatments and, in case of referral, the findings are clearly communicated to medical specialists and backwards.	The contract with GPs is supported by contracts with specialists such as cardiologists and orthopeadic surgeons in which care pathways are stipulated.
8 Prompt access to care	The practice organization of the physician has a number of clinical facilities (e.g., spirometer), daily consultation hours, up-to-date information technology. Patients benefit from shorter waiting times and absence of out-of-pocket payments for medication.	According to the contract practices have to have a comprehensive set of up to date primary care equipment available.
9 Comprehensiveness and care coordination	The physician is trained in primary care-relevant domains (e.g., pain treatment, communication skills) and participates in continuing education.	A committee of the association of GPs and academic departments of general practice in the area coordinates continuing medical education and sets topics for quality circle sessions for the participating GPs together with the AQUA institute.
10 Template of future	Participation in GPCC is a voluntary choice of physicians and patients. For the FP, is associated with about 40% increased reimbursement of the FP for enrolled patients as lump sum payment without pre-specified maximum.	The GP-centred care program is planned to remain in Baden-Wuerttemberg. Collaboration of primary care and medical specialists will be strengthened by programs targeted at ambulatory medical specialists.

In Baden-Wuerttemberg, the largest health insurer (AOK) covers about 40% of the population. For more than a decade, it has consistently developed and implemented programs with financial incentives to strengthen general practice and integration of medical care across sectors and providers. Given its dominant position in the region, this policy has shaped the structure of healthcare in Baden-Wuerttemberg. The programs are largely consistent with the vision of the German College of General Practitioners (DEGAM) and was supported by regional organization of GPs. The support for GP-centred care in other physician organisations was mixed.

Enrollment in the program is a free choice for both patients and physicians. According to regulations for selective contracts in the German Social Code Book Five, the program is based on a contract between the regional sickness fund (‘Allgemeine Ortskrankenkasse’, AOK) and the association of GPs (‘Hausärzteverband’). For the GP, it comes with about 40% additional reimbursement for included patients; there are no direct financial incentives for included patients. An initial evaluation (covering about 1.4 million patients in the first two years) showed that patients enrolled were older and had higher levels of comorbidity than patients in usual care [3]. After adjustment for confounders, enrolled patients showed to have more visits to the GP and fewer visits to medical specialists compared to usual care. Number, costs and appropriateness of medication prescribing had improved, while no impact on rates of hospitalization and rehospitalization rates was found [3].

A major innovation such as this program to strengthen general practice care is likely to be taken up in practice gradually over a long period of time, with most motivated practitioners and the best practice organizations as the first to get involved [4]. Thus, the impact of the program in later days might be lower than in earlier years. Also, the impact in the included patients over a longer period may be different (most likely lower) as compared to patients who have been enrolled for a short time, as easy improvements are likely to have been made in the first period after enrollment. The aim of the present study was to examine the long-term impact of the program to strengthen general practice care.

## Methods

### Study design

This was a comparative evaluation study, based on two separate cross-sectional studies at 4 and 5 years after its start (T1 and T2, respectively). This study was based on data in computerized systems, which are continuously collected for administrative control and reimbursement purposes. Due to legal obligations, the program was offered to all patients and primary care providers, so random allocation to parallel study groups was not possible. Ethical approval for the study was given by the University Hospital Heidelberg Ethics Committee (No. S-359/2013).

### Setting

The project took place in Baden-Wuerttemberg, Germany, a region with about 10.7 million inhabitants. In Germany, approximately 90% of the population is insured by statutory sick funds (the remaining 10% is privately insured). Premiums are payed by employers and employees. Co-payments such as for hospital stays or medication are very low. Data were derived from AOK Baden-Wuerttemberg, the largest health insurer in the region (about 4 million insured persons). In 2015, a total of about 1.4 million patients and about 3.500 primary care physicians participated in the program.

### Study population

Patients were eligible to participate in the evaluation if they met the following pre-defined criteria: aged 18 years or older, living in Baden-Wuerttemberg in the observed year, at least one visit to the primary care physician in the relevant year, health insurance with AOK, no registration with different contracts (e.g., integrated care contracts), no interruptions of registration in the relevant year. Patients enrolled in the program (‘intervention patients’) provided informed consent before participation. They were compared with all other eligible patients in the observed year (‘control patients’). Control patients met the same inclusion criteria as patients in the intervention group. Control patients were linked post-hoc to the primary care physician, whom they had visited in at least 50% of their contacts in primary care. If no such linkage was possible, they were excluded from analysis. The exclusion criteria concerning the contacts to a particular GP was introduced, since for usual care patients, there was no explicit link to their GPs in the data set. Therefore, we determined patients’ GPs on the basis of their GP contacts. Patients without GP contacts and patients that could not be assigned to a particular GP unambiguously were excluded for analyses. The percentage of patients with this exclusion type for both observation periods was 2.1%.

### Program description

The program had multiple components and was targeted at multiple aims. Table 1 maps out the different components of the program against a framework for high-quality primary care [2]. Detailed specifications can be found in the German Social Code, Book Five (SGB V) §73b.

### Measures

Outcomes were chosen in consultation with stakeholders, in particular AOK Baden-Wuerttemberg and Association of GPs (Hausärzteverband). Criteria for selection concerned relevance, changeability, and measurability. More specifically, three questions were considered: a) Does the intervention focus on the target variable directly or maybe indirectly, b) Is the target variable relevant in the Health Services Research context C) Can we expect internal validity for the target variable? An expert panel determined the target variable set. The AQUA Institute for Applied Quality Improvement and Research in Health Care, Göttingen, Germany, has a long term experience in verification and collation of routine data and supported the University of Heidelberg with that experience.

The following 11 outcomes were chosen: number of visits to the primary care physician, the number of patients with polypharmacy (defined as > 5 drug agents), the number of prescriptions that should be avoided on the basis of evidence-based criteria and costs (drugs on a so called ‘red list’), costs of medication therapy in ambulatory care (covering primary and specialist ambulatory care), the number of contacts with medical specialists after referral (with and without referral of the GP), the number of hospital admissions, the number of potentially avoidable hospital admission, the number of days in hospital, the number of hospital admissions within weeks after a previous hospital admission, and costs of hospital admissions.

A list of 107 variables was available for adjustment for confounding of the comparison between groups. The following patient factors were considered as potential predictors based on subject matter knowledge and used for adjustment: patient age, sex, morbidity in observation year (Charlson index [5]), nursing home as place of living, need for nursing (a 4-point scale). GP’s participation in the program was included as well as a number of practice characteristics: urbanization (rural, urban), practice size (number of contacts in relevant period), type of practice (single, group). Data were derived from administrative databases held by AOK. Data-cleaning was performed at the AQUA-institute, Göttingen, independent of the analysis.

### Data-analysis

Given the large sample size, no statistical power calculation was done. Comparison between patients included in the program and non-included patients was done by regression analysis, which took clustering of patients in GPS and GPs in practices into account. Depending on the distribution of each outcome, a linear regression model or a Poisson regression model (for count data) were used. The program effect on each of the outcomes of interest was estimated using adjusted, multivariable regression models. Bonferroni correction for multiple testing was applied for the X estimated program effects. Using a nominal significance level of 5%, the Bonferroni-corrected significance level was 0.05/X and *p* values falling below this level were considered statistically significant.

## Results

Data on 1.187.597 and 1.591.017 eligible patients were available for the analysis for T1 and T2, respectively. Table 2 describes the patient populations. Participants were slightly older than non-participants, but gender, morbidity, and proportion with German nationality were roughly equal in both groups in both years.

**Table 2:** Description of patient samples at T1 (*n* = 1187597) and T2 (*n* = 1591017)

	4 years after start (year 2012)		5 years after start (year 2013)	
	Patients in GPCC (*n* = 610985)	Patients in usual care (*n* = 576612)	Patients in GPCC (*n* = 823951)	Patients in usual care (*n* = 767057)
Mean age in years (SD)	59.3 (17,5)	58.4 (18,3)	58.0 (18.4)	54.9 (19,8)
Women (%)	57.4	58.1	56.6	56.4
German nationality	87.2	86.5	85.7	84.1
Morbidity (mean Charlson index)(SD)	1.5 (2.0)	1.4 (1.9)	1.4 (2.0)	1.1 (1.8)
Stay in GPCC (in quarter years)(SD)	13.2 (2.5)	–	15.6 (4.4)	–

Tables 3 and 4 present the data on outcomes. Compared to usual care, the program was associated with more visits to the FP, more patients with polypharmacy, fewer patients with drugs that should be avoided, and lower medication costs in both years. The number of referrals to ambulatory specialists, either with or without referral from a GP was reduced at T2, but not at T1. In hospital care, the program was associated with fewer hospital admissions and fewer avoidable hospital admissions in both years. Total hospital costs were slightly higher at T1, but lower at T2 for patients in the program. Days in hospital and number of readmissions were lower among patients in the program at T2, but not at T1.

**Table 3:** Impact of the program at T1

	Patients in GPCC	Patients in usual care	Adjusted difference (SE) [95% CI]
Primary care			
Mean number of visits to the FP (SD)	14.32 (11.44)	8.83 (9.83)	*+3.75* (0.177) [3,41; 4,10]
Mean number of prescribed drugs (SD)	5.99 (5.29)	5.85 (5.21)	+0.051 (0.029) [−0.006; 0.108]
Mean percentage of prescriptions that should be avoided per FP (SD)	4.53 (12.43)	5.92 (14.80)	−*1.186* (0.067) [−1.314; −1.051]
Mean costs of medication therapy in ambulatory care in observed year (euro) (SD)	1361.04 (62117.00)	1411.67 (46379.79)	*−113.20* (0.021) [−0.107; −0.054]^a^
Mean number of contacts with medical specialists *with referral from FP (SD)*	3.00 (3.11)	4.06 (4.46)	*−1.01* (0.026) [−1.066; −0.962]
Mean number of contacts with medical specialists *without referral (SD)*	1.93 (2.60)	2.21 (2.90)	*−0.325* (0.02) [−0.365; −0.286]
Hospital care			
Mean number of hospital admissions (SD)	0.272 (0.749)	0.285 (0.774)	*−0.008* (0.008) [−0.044; −0.014]^a^
Mean percentage of avoidable hospital admissions of all admissions (SD)	15.25 (33.15)	16.05 (33.83)	*−1.263* (0.173) [−1.602; −0.923]
Mean number of days in hospital (SD)	13.60 (17.18)	14.04 (18.02)	*−0.008* (0.008) [−0.180; 0.164]
Mean number of hospital admissions within 4 weeks after a previous hospital admission. (SD)	0.200 (0.664)	0.205 (0.692)	−0.004 (0.017) [−0.012; 0.055]
Mean total costs of hospital admission in year (euro) (SD)	5881.59 (8502.35)	5848.46 (8349.58)	*+116.40* (0.021) [0.010; 0.033]^a^

^a^The Standard Error and the 95%-Confidence Interval is reported on the logarithmic scale according to the used link function of the particular model

The italicize figures indicate statistical significance

**Table 4:** Impact of the program at T2

	Patients in GPCC	Patients in usual care	Adjusted difference (SE) (95% CI)
Primary care			
Mean number of visits to the FP (SD)	12.28 (10.85)	8.33 (10.14)	*+1.98* (0.163) [1.659; 2.297]
Mean number of prescribed drugs (SD)	5.99 (5.09)	5.41 (5.14)	+*0.071* (0.022) [0.028; 0.116]
Mean percentage of prescriptions that should be avoided per FP (SD)	2.23 (9.96)	3.20 (11.44)	−*0.699* (0.041) [−0.779; −0.619]
Mean costs of medication therapy in ambulatory care in observed year (euro) (SD)	1371.43 (72.10)	1396.32 (61.72)	*−85.39* (0.009) [−0.064; −0.028]^a^
Mean number of contacts with medical specialists *with referral from FP (SD)*	4.97 (8.50)	4.96 (8.02)	−*0.455* (0.013) [−0.480; −0.428]
Mean number of contacts with medical specialists *without referral (SD)*	2.32 (7.45)	3.42 (9.46)	*−1.528* (0.076) [−1.667; −1.378]
Hospital care			
Mean number of hospital admissions (SD)	0.288 (0.786)	0.290 (0.794)	*−0.017* (0.006) [−0.085; −0.061]^a^
Mean percentage of avoidable hospital admissions of all admissions (SD)	15.69 (33.64)	16.32 (33.96)	*−1.165* (0.145) [−0.880; −1.449]
Mean number of days in hospital (SD)	14.14 (18.13)	14.41 (19.08)	−0.*438* (0.083) [−0.276; −0.599]
Mean number of hospital admissions within 4 weeks after a previous hospital admission. (SD)	0.255 (0.710)	0.233 (0.739)	*−0.007* (0.012) [−0.070; −0.020]
Mean total costs of hospital admission in year (euro) (SD)	6476.29 (9939.46)	6397.86 (10393.27)	*−44.30* (0.003) [−0.017; −0.002]^a^

^a^The Standard Error and the 95%-Confidence Interval is reported on the logarithmic scale according to the used link function of the particular model

The italicize figures indicate statistical significance

## Discussion

Patients who chose to be included in the program of intensified general practice care were compared to patients who chose not to be included, adjusted for a large number of patient characteristics. Four and five years after its start, participation in the program was consistently associated with more intensive use of general practice care, fewer drugs prescribed that should be avoided, lowered medication costs, and fewer hospital admissions. As the program reached a large patient population in routine practice, the benefits for the healthcare system are substantial. We conclude that the program has effectively strengthened general practice care for patients included in the program.

Observational research showed that conditions for providing high-quality chronic care differ widely between practice organisations and between countries [6]. This German program to enhance general practice care is in many ways comparable to medical homes in the United States. A study in 29 practices of the implementation of medical home with shared savings in Pennsylvania, U.S., showed higher rates of primary care visits and lowered rates of ambulatory visits to specialists, fewer emergency department visits, and fewer hospital admissions [7]. These findings are largely similar to the results of our study. The healthcare systems in Germany and the United States share a number of characteristics, such as lack of coordination and the presence financial incentives for overtreatment, so that programs to position GPs in a coordinating role have high chances to achieve improvements in quality and outcomes of healthcare.

The findings of our study are also consistent with macro-level studies, which suggested that patients with chronic diseases receive better care in countries with well-developed primary care. For instance, this was found in an international study that considered aspects of structure, accessibility, continuity, coordination, and comprehensiveness of primary care [8]. All these aspects were targeted by the GP-centred care program. The observation that the proportion of patients with polypharmacy was slightly higher in the program might be explained by a lack of communication between specialists and GPs about prescribing issues. This could lead to overlapping of prescribing with increasing consultation rates.

Our study was based on a large data-set, advanced quantitative and statistical modelling, and included replication of findings by separate analysis on data from two years. It is important to interpret the findings in relation to the study design: we compared patients who chose to be included in the program of intensified general practice care with patients who chose not to be included. Only patients who actually visited the practice in the observed period were included, because this was necessary to link them to a particular GP in this analysis of claims data. Claims about the effectiveness of the program as such have high risk of bias, because we cannot rule out selection bias, despite the extensive adjustment in the multivariate analyses.

In addition, the program comprised of a complex set of interventions (financial incentives, quality circles, databased feedback, software tool with traffic lights system for prescribing drugs, discount drugs) and our study was not designed to unravel the relative impact of its different components or the fidelity of their implementation. Our intention is to analyse trends over longer periods of time in future research, ideally with historical controls (before the program started). The generalizability may be limited by its focus on Baden-Wuerttemberg, a relatively wealthy state in Germany. However, people insured by AOK tend to be less wealthy, so we think that the generalizability is reasonably good.

Future research should include process evaluation, which explores the added value of different components of the program. The added payment seems crucial to get a large number of healthcare providers involved in the program, but it does not improve quality or outcomes of healthcare by itself. Healthcare providers have responded to these financial incentives, but how exactly is largely unclear. Organizing healthcare delivery in a more structured way has stimulated that patients receive all items of recommended healthcare. A weaker aspect of the GP-centred care program is the degree of support for patients’ self-management of health and disease, as this mainly comprises of traditional patient education. The overall impact on health outcomes may be higher, if modern tools to enhance patient self-management would be included.

The study supports the policy of various stakeholders in Baden-Wuerttemberg to strengthen primary care. Although attribution of outcomes to interventions should be carefully done, the routine care setting and size of the study lend support to this claim. It may be noted that primary care in Germany is moderately strong, like in many parts of the United States. The generalizability may be best to countries, which are similar to Germany regarding primary care. Future research should also focus on in-depth analysis of the added value of different components of the program and on its impact on clinical processes and outcomes. Nevertheless, the study suggests that a comprehensive package of interventions can effectively strengthen general practice care.

## References

[1] Primary care physician contract. http://www.hausarzt-bw.de/upload/AOK%20Vertragsunterlagen/Vertrag_295a_KJ__Stand_01.01.2014.pdf. (Accessed: 22 Dec 2015).

[2] Bodenheimer T, Ghorob A, Willard-Grace R, Grumbach K (2014). The 10 building blocks of of high-performing primary care. Ann Fam Med.

[3] Laux G, Kaufmann-Kolle P, Bauer E, Goetz K, Stock C, Szecsenyi J (2013). Evaluation of family-doctor centred medical care based on AOK routine data in Baden-Wuerttemberg. Z Evid Fortbild Qual Gesundh wesen.

[4] Rogers EM (2003). Diffusion of innovation.

[5] Sundararajan V, Henderson T, Perry C, Muggivan A, Quan H, Ghali WA (2004). New ICD‐10 version of the Charlson comorbidity index predicted in‐hospital mortality. J Clin Epidemiol.

[6] Van Lieshout J, Goldfracht M, Campbell S, Ludt S, Wensing M (2011). Primary care characteristics and population-orientated health care across Europe: an observational study. Br J Gen Pract.

[7] Friedberg MW, Rosenthal MB, Werner RM, Volpp KG, Schneider EC (2015). Effects of a medical home and shared savings intervention on quality and utilization of care. JAMA Intern Med.

[8] Hansen J, Groenewegen PP, Boerma WGW, Kringos DS (2015). Living in a country with a strong primary care system is beneficial for to people with chronic conditions. Health Aff.

